# RNA Interference Depletion of the Halloween Gene *Disembodied* Implies its Potential Application for Management of Planthopper *Sogatella furcifera* and *Laodelphax striatellus*

**DOI:** 10.1371/journal.pone.0086675

**Published:** 2014-01-28

**Authors:** Pin-Jun Wan, Shuang Jia, Na Li, Jin-Mei Fan, Guo-Qing Li

**Affiliations:** Education Ministry Key Laboratory of Integrated Management of Crop Diseases and Pests, College of Plant Protection, Nanjing Agricultural University, Nanjing, China; Natural Resources Canada, Canada

## Abstract

*Sogatella furcifera* and *Laodelphax striatellus* are economically important rice pests in China by acting as vectors of several rice viruses, sucking the phloem sap and blocking the phloem vessels. Ecdysteroid hormone 20-hydroxyecdysone regulates insect development and reproduction. A cytochrome P450 monooxygenase CYP302A1 (22-hydroxylase), encoded by the Halloween gene *disembodied* (*dib*), plays a critical role in ecdysteroidogenesis. The objective of this study is to test whether *dib* genes are potential targets for RNA interference-based management of *S. furcifera* and *L. striatellus.* We cloned and characterized *Sfdib* and *Lsdib*. The open reading frame regions of *dib* genes were generated and used for designing and constructing dsRNA fragments. Experiments were conducted using oral delivery of ds*dib* to investigate the effectiveness of RNAi in *S. furcifera* and *L. striatellus* nymphs. Real-time quantitative reverse transcriptase-PCR analysis demonstrated that continuous ingestion of ds*dib* at the concentration of 0.01, 0.05 and 0.50 mg/ml diminished *Sfdib* expression levels by 35.9%, 45.1% and 66.2%, and *ecdysone receptor* (*SfEcR*) gene mRNA levels by 34.0%, 36.2% and 58.5% respectively in *S. furcifera*, and decreased *Lsdib* expression level by 18.8%, 35.8% and 56.7%, and *LsEcR* mRNA levels by 25.2%, 46.8% and 68.8% respectively in *L. striatellus*. The reduction in *dib* and *EcR* transcript abundance resulted in observable phenotypes. The development of nymphs was impaired and the survival was negatively affected. Our data will enable the development of new insect control strategies and functional analysis of vital genes in *S. furcifera* and *L. striatellus* nymphs.

## Introduction

The white-backed planthopper *Sogatella furcifera* and the small brown planthopper *Laodelphax striatellus* are economically important pests of rice in China. They cause serious damage to rice plants by acting as viral vectors, and by sucking the phloem sap and blocking the phloem vessels. In China, *S. furcifera* and *L. striatellus* are found in all rice-growing areas, especially in the middle and downstream Yangtze River. The most common management strategy to control these planthoppers is chemical treatments. However, this inevitably leads to the development of insecticide resistance, insect resurgence, and serious environmental pollution [1]–[5]. Alternative control strategies must be developed.

Among the potential control strategies, RNA interference (RNAi), an RNA-dependent gene silencing process induced by double-stranded RNA (dsRNA), has been proposed as a promising approach to control the damage caused by insect pests [6], [7]. In the brown planthopper *Nilaparvata lugens*, for example, dietary introduction of dsRNAs can not only effectively silence target genes that are mainly expressed in gut [8]–[10], or widely expressed in various tissues [11], [12], but also knock down target genes which are expressed in other tissues far from the midgut [13]. Similar results have been documented in *S. furcifera* and *L. striatellus* [14]–[16]. These results demonstrate that RNAi has great potential in planthopper management. Identification of the most attractive candidate genes for RNAi in *S. furcifera* and *L. striatellus* is the first step.

Ecdysteroid 20-hydroxyecdysone (20E) regulates insect development and reproduction. A cytochrome P450 monooxygenase CYP302A1 (22-hydroxylase), encoded by the Halloween gene *disembodied* (*dib*), has been confirmed to be involved in ecdysteroidogenesis in two dipteran species *Drosophila melanogaster* and *Anopheles gambiae*, and two lepidopteran species *Bombyx mori* and *Manduca sexta*, by expression of their corresponding genes in S2 cells [17]–[22]. Moreover, in *D. melanogaster*, several mutants in *dib* gene have been found [17]. Among them, *dib^F8^* and *dib^P3^* produce truncated proteins that lack the heme binding site. The *dib^13A^* allele is characterized by a premature stop codon that removes the last five amino acids [18]. Analysis by RP-HPLC/RIA demonstrate that the homozygous *dib* mutant embryos have very low titers of ecdysone (E) and 20E and fail to express *IMP-E1* and *L1*, two 20E-inducible genes, in certain tissues of the embryo. Phenotypic analysis reveals that *dib* mutant embryos produce little or no cuticle, exhibit severe defects in many late morphogenetic processes such as head involution, dorsal closure and gut development, and finally die at the embryonic stage [18]. Furthermore, mostly based on sequence similarity, *dib* genes are described or predicted in hemipteran *Acyrthosiphon pisum* [23] and *Bemisia tabaci* [24], in dipteran *Aedes aegypti* [25], in lepidopteran *Spodoptera littoralis* [26], and in hymenopteran *Apis mellifera* [27]. In addition to insects, a *dib* gene has been identified in the water flea *Daphnia pulex* [28].

In the present paper, we cloned and characterized *dib* genes in *S. furcifera* and *L. striatellus*. We demonstrated the insecticidal action of orally-delivered dsRNA (ds*dib*) in *S. furcifera* and *L. striatellus* nymphs. We found that continuous ingestion of ds*dib* delayed nymphal growth and caused lethality in both planthopper species. Our data will enable the development of new insect control strategies and functional analysis of vital genes.

## Materials and Methods

### Insect Culture

*S. furcifera* and *L. striatellus* were routinely reared on the rice (*Oryza sativa*) variety Taichung Native 1, using a protocol described recently [14], [15]. In our laboratory, *S. furcifera* eggs hatched into nymphs within 7 days. Nymphs went through 5 instars, with the first-, second-, third-, fourth- and fifth-instars lasting 2.5, 2.0, 2.0, 3.0 and 3.0 days, respectively. *L. striatellus* eggs hatched into nymphs within 13 days. The first-, second-, third-, fourth- and fifth-instar nymphs spent an average of 6.0, 5.0, 5.0, 5.0 and 6.0 days, respectively.

### Cloning and Sequencing of Full-length *Lsdib* cDNA

We obtained *Sfdib* and *Lsdib* unigenes from the corresponding transcriptome datasets of *S. furcifera* and *L. striatellus* recently constructed in our laboratory [14], [15]. The resulting sequences were authenticated by polymerase chain reaction (PCR) using specific primers (Table 1), the same thermal cycling conditions and PCR reaction system as described recently [14], [15]. The full-length *Sfdib* and *Lsdib* cDNAs were obtained by 5′- and 3′-RACE, using antisense and sense gene-specific primers (Table 1) and the universal primers in the SMART™ RACE kit, and the standard manufacturer-recommended components and thermal cycling conditions. The PCR products were then cloned into pGEM-T easy vector (Promega), and sequenced at both strands. After full-length cDNAs were obtained, two primer pairs were designed (Table 1) to verify the sequence. Open reading frames were predicted using the editseq program of DNAStar (http://www.dnastar.com). The nucleotide sequences were finally annotated by Dr. David Nelson in accordance with the CYP nomenclature committee convention (http://drnelson.uthsc.edu/cytochromeP450.html) [29]. The resulting sequences were submitted to GenBank (*Sfdib*, KC579455; *Lsdib*, KC579460).

**Table 1:** Primers used in RT-PCR, 5′ and 3′ RACE, synthesizing dsRNA, and performing qRT-PCR.

Primer	Sequence (5′ to 3′)	Amplicon size (bp)
Primers used in RT-PCR		
SfdibFp	GCCCAGGTCCTTCTCGTT	300
SfdibRp	GGTCCGTTTGATGGCAGA	
LsdibFp	AAATGGGGAACCGATAGTGT	1431
LsdibRp	TTTATACCGACGGCAGAGC	
Primers used in 5′-RACE		
SfdibGSP	ATTCCGATTGCCACCGGGTTAA	
SfdibNGSP	TCGGGATGTTTGGAAAGGTTGTGC	
LsdibGSP	TAGCACCCTTCCAATGCCGATG	
LsdibNGSP	CGGGGTGTCTGGAAAGGTTGTGC	
Primers used in 3′RACE		
SfdibGSP	TGGCAATCGGAATCGGAAGGGTG	
SfdibNGSP	ACGCTTCTTCCGCAACGAGGGC	
LsdibGSP	TCAGCGATGCCGAACTTCACCC	
LsdibNGSP	GTCTCGCAGAACCAAGTGGCCTGTC	
Primers used in PCR for End to End		
SfdibFp	GATTGGATCGCCCTTAA	2888
SfdibRp	ATAGCCTATGCTACTCTAACG	
LsdibFp	GTGAAATCGGTGATCTCATT	1682
LsdibRp	TTTACGTTCAGTAAACTTG	
Synthesizing the dsRNAs		
SfdibFd	taatacgactcactataggg TCGAGGAAGTTGCACTG	458
SfdibRd	taatacgactcactataggg ACAGGCGACTTGGTTCT	
LsdibFd	taatacgactcactataggg ACATCATTGGTCGCATTC	406
LsdibRd	taatacgactcactataggg TGTAGCTCATACTGGAGGTTAT	
egfpup	taatacgactcactataggg AAGTTCAGCGTGTCCG	414
egfpdown	taatacgactcactataggg CTTGCCGTAGTTCCAC	
Performing the qPCR		
SfdibFq	TCGGACAAATCGTGAGGGAAGAC	116
SfdibRq	GACTGTAGCGTTGCGGGTAGG	
SfEcRFq	AATGAGTTCGAGCACCCTAGCGAA	129
SfEcRRq	AATGGTGATTTCGGTGATGTGGCG	
SfRPL9Fq	TGTGTGACCACCGAGAACAACTCA	131
SfRPL9Rq	ACGATGAGCTCGTCCTTCTGCTTT	
SfARFFq	CACAATATCACCGACTTTGGGATTC	141
SfARFRq	CAGATCAGACCGTCCGTACTCTC	
LsdibFq	ATTGCTAAGAAGGTTGCTGAACTCG	132
LsdibRq	TGGTGTATCTGGCTGGTGAACG	
LsEcRFq	AAACTGTTGCGCGAGGACCAAATC	187
LsEcRRq	AGAACTGCAACAGGTCTTCGACCA	
LsEF-1Fq	CCTTACCCATGTTGGATGCTTATT	95
LsEF-1-Fq	TGCTTCTGTCTTCCTCTTTCTTCC	
LsARFFq	TTGGACAGTATCAAGACCCATC	104
LsARFRq	GCAGCAATGTCATCAATAAGC	

### Multiple Sequence Alignment and Phylogenetic Analysis

The annotated Dib proteins from a spider species *Tetranychus urticae* and 17 insect species were aligned with the predicted SfDib and LsDib sequences using ClustalW2.1 [30]. The alignment was used to construct the maximum-likelihood (ML) trees using RAxML version 7.26 [31] to select the best-fitting model (LG+γ) after estimation by ProtTest [32]. The reliability of the ML tree topology was evaluated by bootstrapping a sampling of 1000 replicates.

### Bioassays Using dsRNA, 20E and their Mixtures

DNA samples for the production of *Sfdib*, *Lsdib* and enhanced green fluorescent protein (*egfp*, control) were synthesized by PCR, using a 458 bp *Sfdib* cDNA, a 406 bp *Lsdib* cDNA and a 414 bp *egfp* fragments, and gene-specific primers (Table 1) incorporating the T7 RNA polymerase promoter sequence (5′-taatacgactcactataggg-3′) [14], [15]. PCR products were purified using the Wizard H SV Gel and PCR Clean-Up System (Promega, Madison, WI, USA) and used for dsRNA synthesis using the T7 Ribomax TM Express RNAi System (Promega, Madison, WI, USA). The synthesized dsRNAs were respectively isopropanol-precipitated, resuspended in nuclease-free water, quantified by a spectrophotometry (NanoDrop TM 1000, Thermo Fisher Scientific, USA) at 260 nm, and kept at −70°C until use.

A dietary dsRNA-introducing procedure previously reported [8], [13] was used to feed the insects, with glass cylinders (12 cm in length and 2.8 cm in internal diameter) as feeding chambers. Twenty *S. furcifera* first-instar nymphs or *L. striatellus* third-instar nymphs were carefully transferred into each chamber and pre-reared for one day to the next instar, on liquid artificial diet between two layers of stretched Parafilm M (Pechiney Plastic Packaging Company, Chicago, IL, USA) that were placed at both ends of the chamber. The artificial diet containing one of the dsRNAs was then used to feed the nymphs. The diet was changed and dead nymphs were removed daily.

Two bioassays were carried out for each planthopper species, with its corresponding ds*dib* (ds*Sfdib* for *S. furcifera* nymphs, ds*Lsdib* for *L. striatellus* nymphs). The first experiment had five treatments including non-dsRNA diet (blank control), the diet containing ds*egfp* at the concentration of 0.50 mg/ml (negative control), and the diets containing ds*dib* at the concentration of 0.05, 0.01 and 0.50 mg/ml. The second bioassay was a rescue experiment. 20E (Sigma, purified by reverse-phase HPLC before experiments) in ethanol was mixed with the diet to obtain a final concentration of 0.50 mg/ml diet, a dose that has no obvious negative effects on planthopper species [14], [15]. There were four treatments: non-dsRNA+ethanol diet (blank control), 0.50 mg/ml of ds*egfp*+ethanol diet (negative control), 0.50 mg/ml of ds*dib*+ethanol diet, and 0.50 mg/ml ds*dib*+0.5 mg/ml of 20E diet. All treatments in the experiments were replicated 25 times (25 chambers), and a total of 250 nymphs in each treatment were used (100 nymphs for bioassays and 150 nymphs for q-PCR).

The experiments lasted 6 days for *S. furcifera*, and 5 days for *L. striatellus* nymphs. Mortality was recorded daily. After each experiment, the surviving nymphs were transferred to rice to measure the development delay. The instars were identified by the same method described recently [14], [15]. Mortalities from the termination of experiment to adults emergence were also calculated.

### Real-time Quantitative PCR

For *S. furcifera*, total RNA samples were prepared from whole bodies of the second-instar (Day 1, 2 in the second instar, I2D1 and I2D2), third-instar (I3D1 and I3D2) and fourth-instar (I4D1, I4D2 and I4D3) nymphs on rice, from the head, thorax and abdomen of I4D3 nymphs, and from nymphs subjected to the 6-day bioassays. For *L. striatellus*, total RNA samples were prepared from I4D1 to I4D5, and I5D1 nymphs on rice, from the head, thorax and abdomen of I4D5 nymphs, and from whole bodies of nymphs which had been exposed to dsRNA for 5 days. Each sample contained 5–10 nymphs and repeated in biological triplicate. All RNAs were isolated using SV Total RNA Isolation System Kit (Promega). The expression levels of *Sfdib* and *SfEcR* in each *S. furcifera* nymphal sample were measured by qRT-PCR as described proviously [14], [15]. The *S. furcifera ADP-ribosylation factor* (*ARF*) and *ribosomal protein RPL9* were used as internal controls. The mRNA abundance of *Lsdib* and *LsEcR* in each *L. striatellus* nymphal sample was measured using the internal control genes *ADP-ribosylation factor* (*ARF*) and *elongation factor-1* (*EF-1*) (primers for these genes are listed in Table 1). Each sample was repeated in technical triplicate. Data were analyzed by the 2^−ΔΔCt^ method [33], using the geometric mean of the two internal control genes for normalization according to the strategy described previously [33], [34].

### Data Analysis

The data were given as means ± SE, and were analyzed by ANOVA or a repeated measures ANOVA followed by the Tukey-Kramer test, using SPSS for Windows (SPSS, Chicago, IL, USA).

## Results

### Molecular Cloning and Sequence Analysis

**Figure 1:**
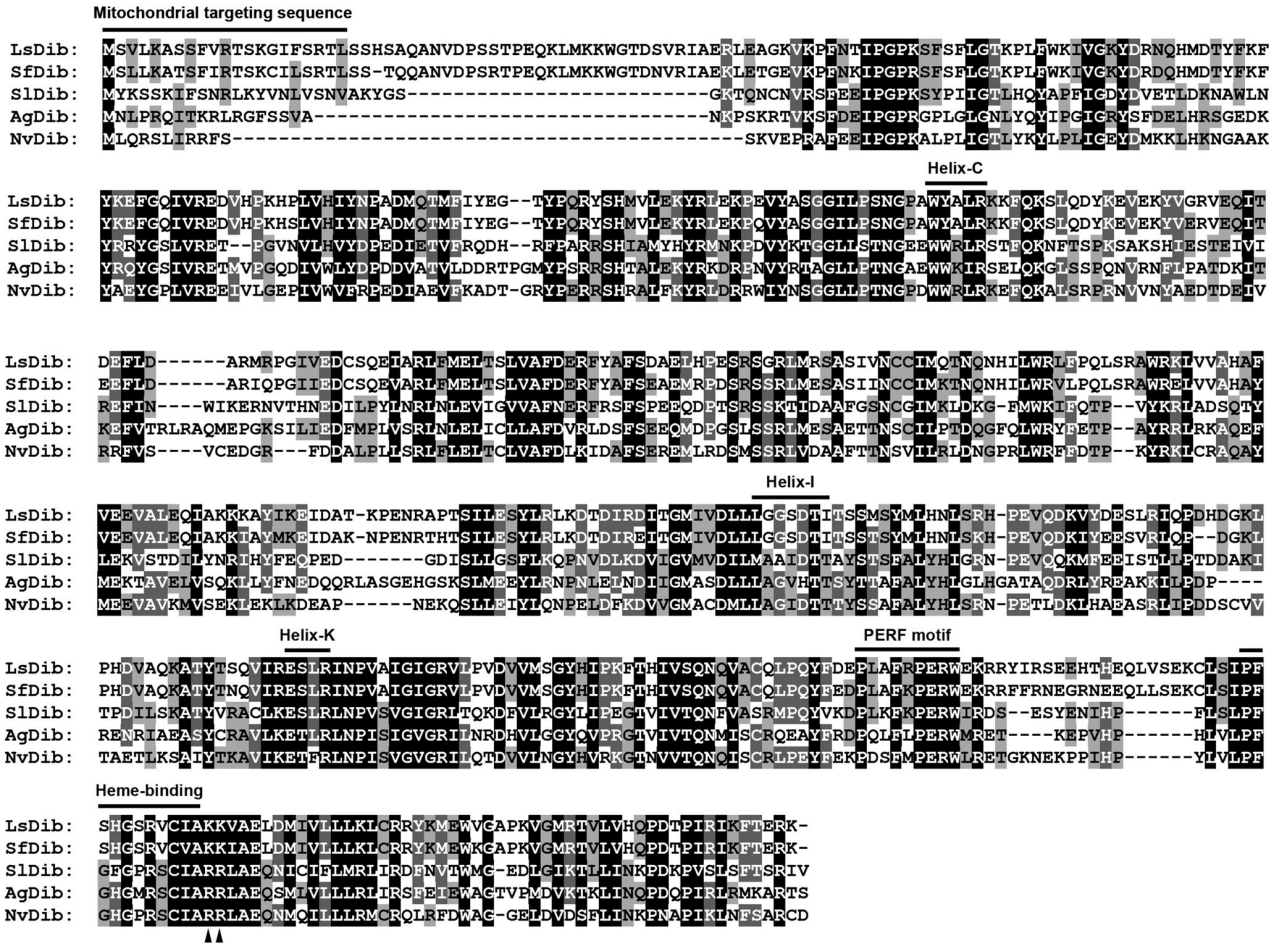
Amino acid sequence alignment of Dib-like proteins. ClustalX (2.1) multiple sequence alignment program were used. CYP302A1 (Disembodied, Dib) originates from *Spodoptera littoralis* (SlDib, ACM46003), *Anopheles gambiae* (AgDib, NP_524810), *Nasonia vitripennis* (NvDib, XP_001601675), *Sogatella furcifera* (SfDib) and *Laodelphax striatellus* (LsDib) respectively. Amino acids with 100%, >80%, and >60% conservation are shaded in black, dark grey and light grey, respectively. The characteristic P450 structure, a mitochondrial targeting segment, Helix C, Helix I, Helix K, PERF motif and Heme-binding domain are indicated above the alignment. Two positively charged residues, a signature for mitochondrial enzyme, are indicated with triangles.

Two cDNAs of the putative Halloween gene *dib* (*disembodied*, *cyp302a1*) were obtained. *Sfdib* from *S. furcifera* contained a complete coding sequence and encoded a 547-amino-acid protein. Similarly, *Lsdib* from *L. striatellus* had a complete open reading frame which encoded for a putative 22-hydroxylase of 550 amino acid residues (Fig. 1).

The deduced amino acid sequences of SfDib and LsDib share 95% identity. Moreover, SfDib and LsDib have 30% and 30%, 57% and 55%, 56% and 55%, and 54% and 54% of identities with the homologues from *A. pisum*, *Tribolium castaneum*, *M. sexta* and *D. melanogaster*. Both SfDib and LsDib have five conserved motifs typical of insect P450s, i.e., WxxxR (Helix-C), GxE/DTT/S (Helix-I), ExxR (Helix-K), PxxFxPE/DRF (PERF motif) and PFxxGxRxCxG/A (heme-binding domain), where ‘x’ means any amino acid [35], [36] (Fig. 1).

Insect Dib belongs to the mitochondrial P450 family. The N-termini of SfDib and LsDib have a typical mitochondrial targeting sequence, consisting of several charged residues. Moreover, both SfDib and LsDib possess two positive charged residues near heme-binding domain, another character of typical mitochondrial P450 enzymes (Fig. 1).

**Figure 2:**
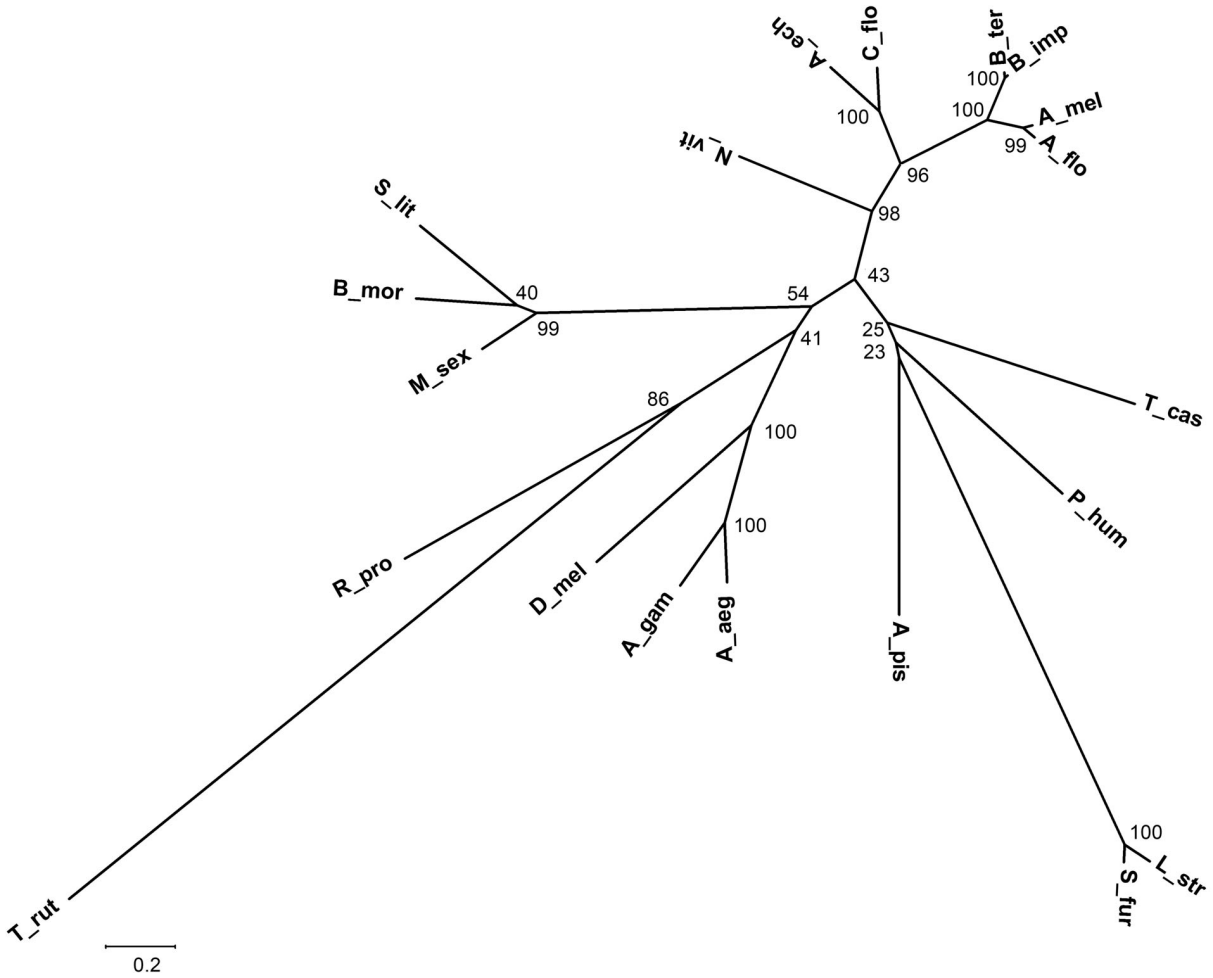
A phylogenetic tree of Dib-like proteins from representative insect species. An unrooted phylogenetic tree was constructed by the maximum-likelihood method based on the protein sequence alignments. Dib-like proteins from the spider mite *Tetranychus urticae* (T_urt, JGI_V11_204789), and 19 insect species are shown. *Bombus terrestris* (B_ter, XP_003394738), *B. impatiens* (B_imp, XP_003492183), *Apis florea* (A_flo, XP_003695126), *A. mellifera* (A_mel, XP_001122832), *Camponotus floridanus* (C_flo, EFN63712), *Acromyrmex echinatior* (A_ech, EGI60082), *Nasonia vitripennis* (N_vit, XP_001601675), *Anopheles gambiae* (A_gam, ABU42523), *Aedes aegypti* (A_aeg, AAX85205), *Drosophila melanogaster* (D_mel, NP_524810), *Manduca sexta* (M_sex, ABC96069), *Bombyx mori* (B_mori, NP_001036953), *Spodoptera littoralis* (S_lit, ACM46003), *Tribolium castaneum* (T_cas, XP_974252), *Pediculus humanus* (P_hum, XP_002426284), *Acyrthosiphon pisum* (A_pis, XP_001948299), *Rhodnius prolixus* (*R_pro*, *RPRC011595-RA*), *Sogatella furcifera* (S_fur) and *Laodelphax striatellus* (L_str), respectively. The percentiles of bootstrap values (1,000 replicates) are indicated. The scale bar represents the amino acid divergence.

The evolutionary relationship of Dib-like proteins derived from a spider mite species and 19 insect species was evaluated (Fig. 2). Those from 19 insects were distant from that came from the spider mite *Tetranychus urticae*. In 19 insect species, the Dib-like proteins formed a Hymenoptera clade, a Diptera clade and a Lepidoptera clade. Among the Hymenopteran clade, three sub-clades were formed by proteins derived from Apidae, Formicidae and Pteromalidae, supported by 100% 96% and 98% of bootstrap value respectively. In the Apidae sub-clade, two Dibs from the genus *Bombus* (B_ter and B_imp) and two from the genus *Apis* (A_mel and A_flo) clustered together, supported by 100% and 99% of bootstrap values. Among the Dibs of the dipteran clade, two from *A. aegypti* (A_aeg) and *A. gambiae* (A_gam) formed a mosquito sub-clade with 100% bootstrap support, and then the mosquito sub-clade and that from *D. melanogaster* (D_mel) joined together to form the dipteran clade, supported by 100% of bootstrap values. However, four Dib-like proteins from Hemiptera formed two clades. Three putative Dib proteins from *S. furcifera* (S_fur), *L. striatellus* (L_str) and *A. pisum* (A_pis) formed one clade, which was well segregated from a putative *Rhodnius prolixus* Dib. Moreover, the placements of Dibs from *T. castaneum* (T_cas) and *Pediculus humanus* (P_hum) were also not well consistent with the traditional view of insect phylogeny (Fig. 2).

### Temporal and Spatial Transcript Profiles

**Figure 3:**
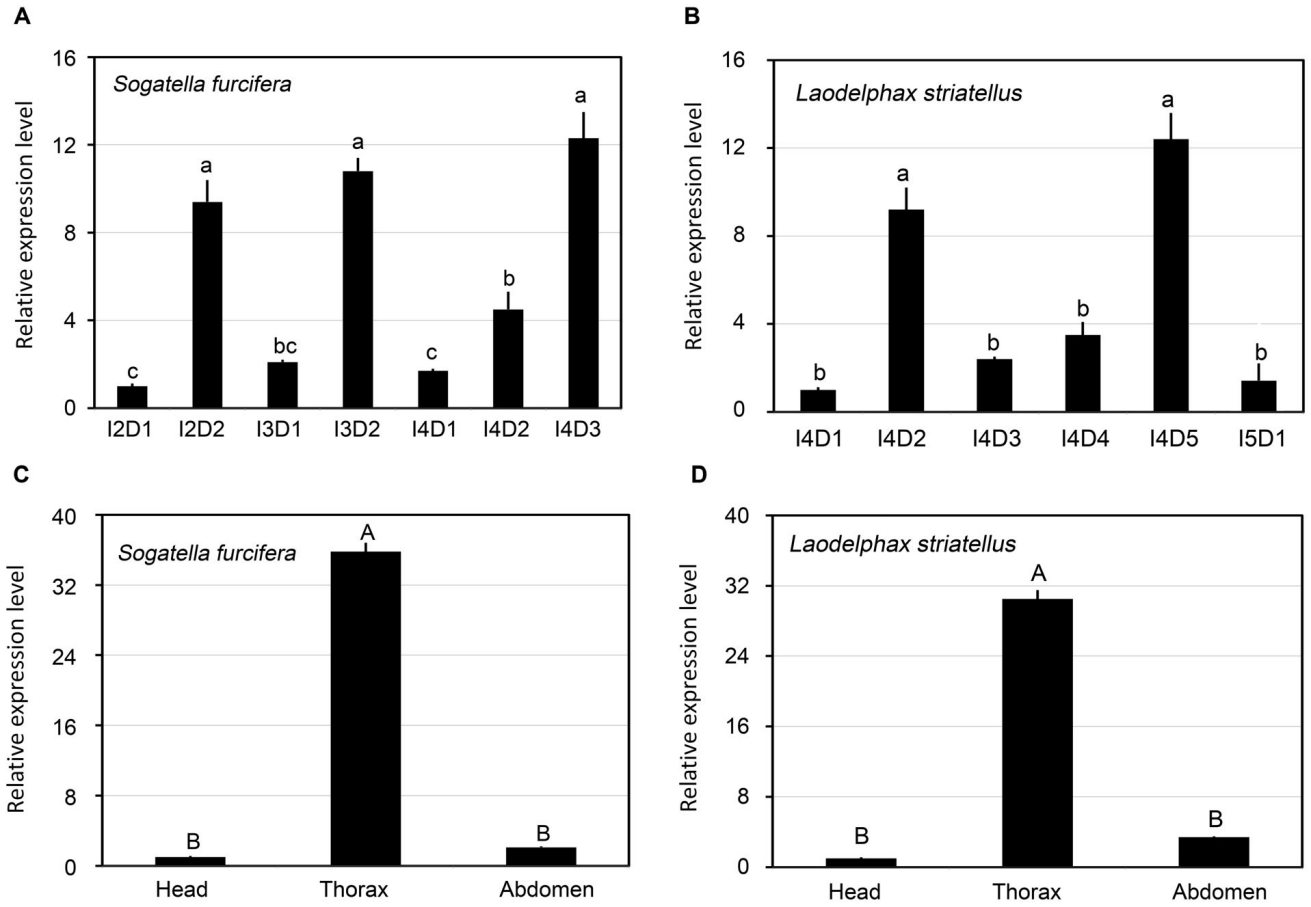
Relative transcript levels of *dib* genes. The mRNA levels were measured in *S. furcifera* second through fourth instars (I2D1, I2D2, I3D1, I3D2, I4D1, I4D2 and I4D3) (A) and in *L. striatellus* fourth (I4D1 to I4D5) and fifth instars (I5D1) (B) at 24 h intervals, and in the head, thorax and abdomen of *S. furcifera* I4D3 (C) and *L. striatellus* I4D5 (D) nymphs. For each sample, 3 independent pools of 5–10 nymphs were measured in technical triplicate using qRT-PCR. The values were calculated using the 2^−ΔΔCt^ method. The relative expression levels were the ratios of relative copy numbers in individuals of specific developmental stage or specific body part to that in *S. furcifera* I2D1, *L. striatellus* I4D1 or the head of corresponding species. The bars represent averages with vertical lines indicating SE. Bars with the same lowercase or uppercase letters are not statistically different at P = 0.05 or 0.01.

For *S. furcifera*, second-, third- and fourth-instar nymphs lasted an average of 2.0, 2.0 and 3.0 days. *Sfdib* showed three expression peaks at day 2 of the second-instar (I2D2), day 2 of the third-instar (I3D2) and day 3 of the fourth-instar nymphs (I4D3). In contrast, the expression levels were lower in the newly-molted second (I2D1)-, third (I3D1)- and fourth (I4D1)-instar nymphs. An ANOVA analysis (P<0.05, df = 6,14) showed that the three peaks were significantly higher than the three troughs (Fig. 3A).

In our rearing protocol, *L. striatellus* fourth-instar nymphs lasted an average of 5.0 days. *Lsdib* showed two expression peaks, with the first at day 2 and the second at day 4–5 in fourth-instar nymphs. In contrast, the expression levels were lower and formed two troughs in the newly-molted fourth- and fifth-instar nymphs. An ANOVA analysis (P<0.05, df = 5,12) revealed that the two peaks were significantly higher than the two troughs (Fig. 3B).

Figs. 3C and 3D showed the spatial distribution of *Sfdib* and *Lsdib* on day 3 and day 5 of *S. furcifera* and *L. striatellus* fourth-instar nymphs. Both *Sfdib* and *Lsdib* are expressed at a higher level in the thoraces where prothoracic glands were located. Moreover, trace amounts of transcripts were found in the heads and abdomens. An ANOVA analysis (P<0.01, df = 2,6) demonstrated that the expression levels in thoraces were significantly higher than the mRNA levels in heads and abdomens.

### Effect of Dietary Introduction of dsRNA on the Expression of *dib* and *EcR* Genes

**Figure 4:**
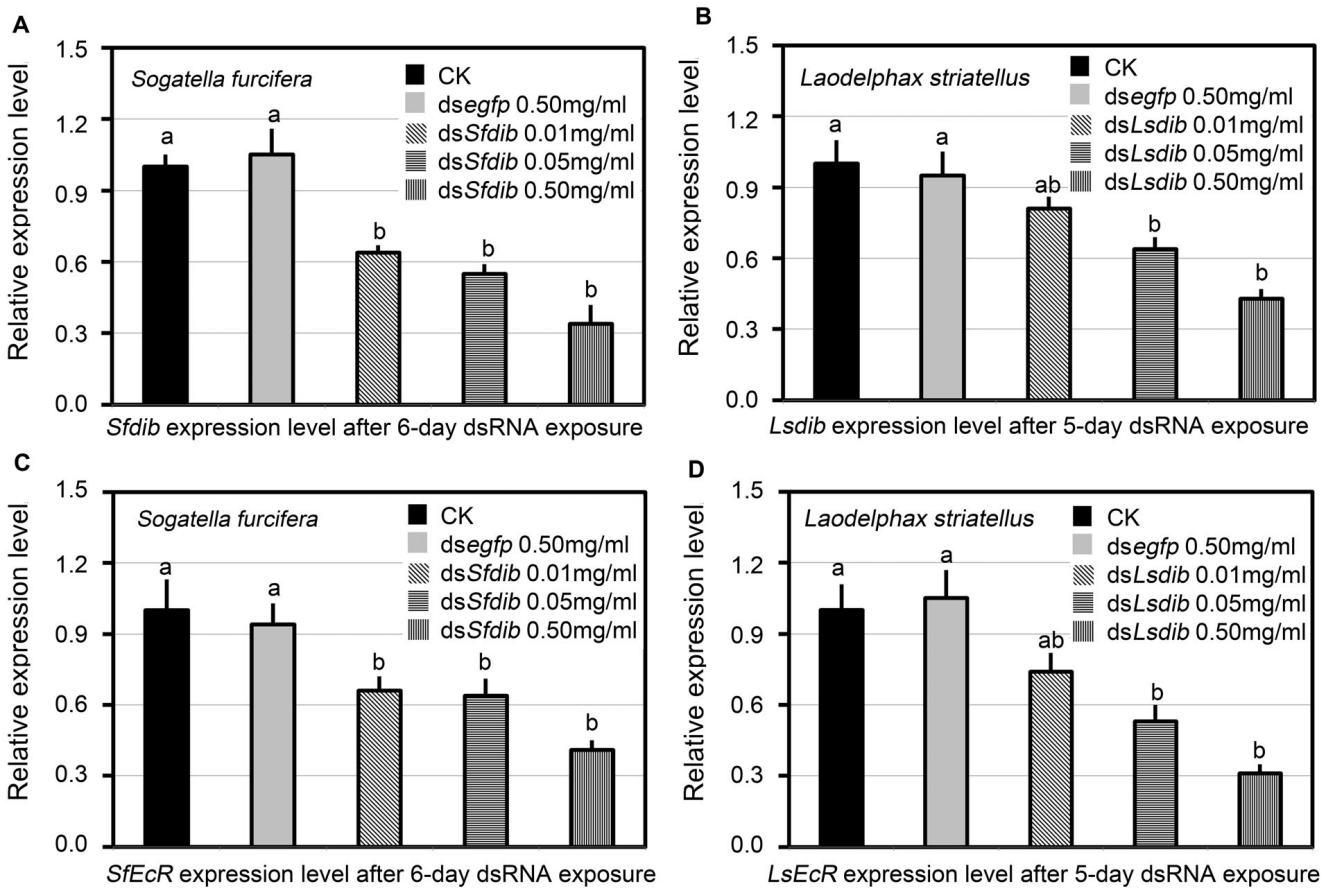
Relative expression levels of *dib* (A and B) and *EcR* (C and D) genes. The mRNA levels were measured in *S. furcifera* and *L. striatellus* after continuously exposed to dietary dsRNA at different concentrations. For each sample, 3 independent pools of 5–10 nymphs were measured in technical triplicate using qRT-PCR. The values were calculated using the 2^−ΔΔCt^ method. The relative expression levels were the ratios of relative copy numbers in individuals ingested dsRNA to that in blank control. The bars represent averages with vertical lines indicating SE. Bars with the same lowercase letters are not statistically different at P = 0.05.

Dietary introduction of ds*dib* reduced *dib* transcript levels. In *S. furcifera*, a 6 day period of continuous ingestion of the diet containing ds*Sfdib* at the concentration of 0.01, 0.05 and 0.50 mg/ml diminished *Sfdib* expression level by 35.9%, 45.1% and 66.2% respectively. The three expression levels were significantly lower than those in the blank control (Fig. 4A). Similarly, after 5-day ingestion of ds*Lsdib* at the concentration of 0.01, 0.05 and 0.50 mg/ml, the mRNA levels of *Lsdib* in treated *L. striatellus* nymphs were reduced by 18.8%, 35.8% and 56.7% respectively, with the last two significantly lower than that the blank control (Fig. 4B). In contrast, *Sfdib* and *Lsdib* expression levels in ds*egfp*-exposed planthoppers were not changed (Fig. 4A and 4B).

Since Dib is expected to affect the expression of genes involved in the ecdysteroid-signaling pathway, the effects of *Sfdib* and *Lsdib* knockdown were examined on the transcript levels of *ecdysone receptor* genes *SfEcR* and *LsEcR*. EcR constitutes one of the two subunits of the 20E heterodimeric nuclear receptor. EcR is also known to be regulated by 20E through a positive feedback loop directly [37] or indirectly [38] in *D. melanogaster*. As expected, *SfEcR* expression levels in *S. furcifera* nymphs that had ingested ds*Sfdib* at the concentration of 0.01, 0.05 and 0.50 mg/ml significantly decreased by 34.0%, 36.2% and 58.5% respectively, when compared with that in blank control (Fig. 4C). Similarly, *LsEcR* expression levels in *L. striatellus* nymphs that had ingested ds*Lsdib* at the concentration of 0.01, 0.05 and 0.50 mg/ml reduced by 25.2%, 46.8% and 68.8% respectively, with the last two concentrations significantly lower than that in blank control (Fig. 4D). In contrast, *Sfdib* and *Lsdib* expression level in ds*egfp*-fed planthoppers was not different than insects in the blank control (Fig. 4C and 4D).

### Effects of Ingestion of dsRNA on Nymph Survival

**Figure 5:**
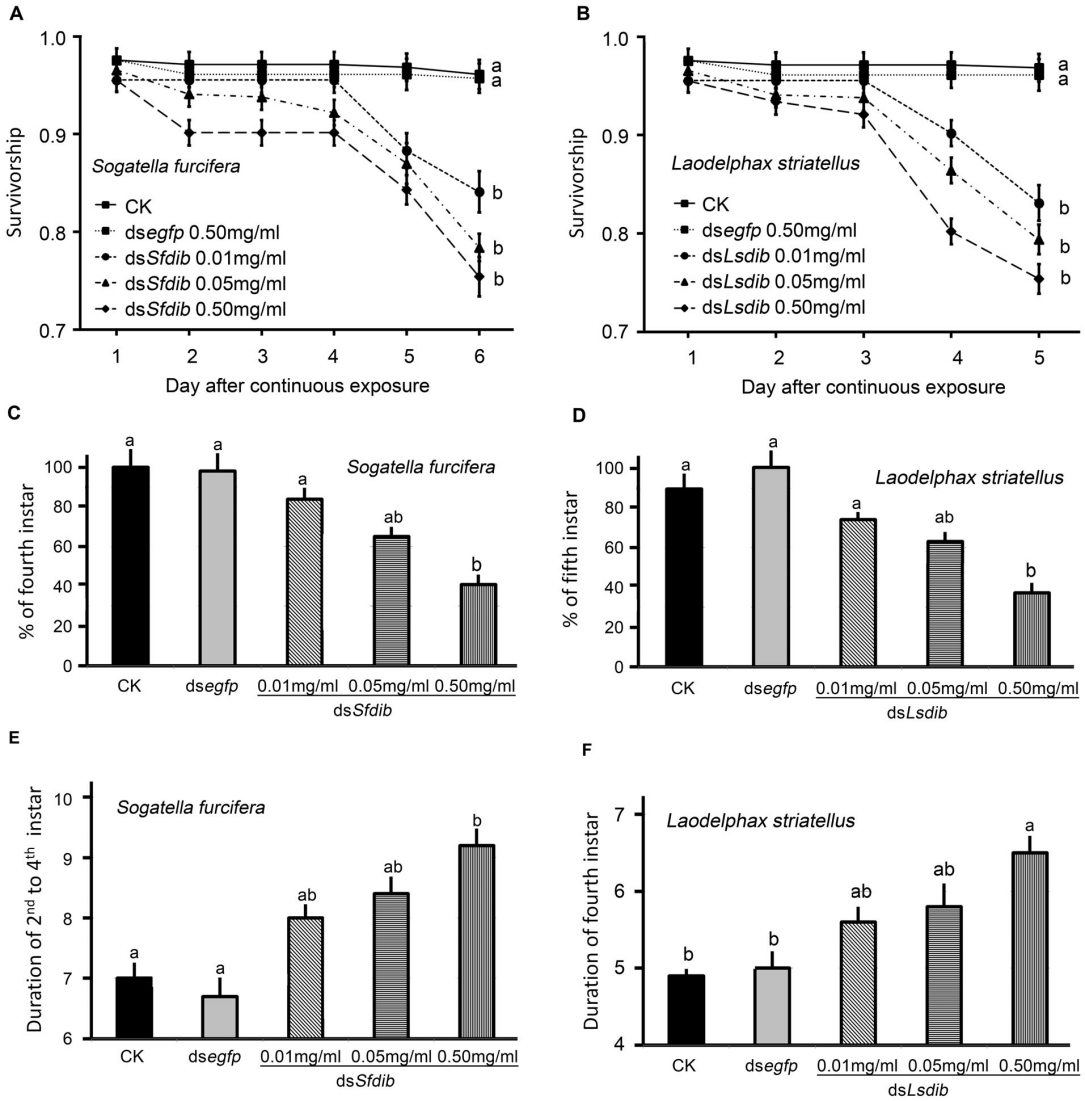
Survival (A, B) and duration (C, D) of *S. furcifera* and *L. striatellus*. The nymphs were continuously exposed to dsRNA at different concentrations through the fourth-instar stage. The values represent averages with vertical lines indicating SE. Values with the same lowercase letters are not statistically different at P = 0.05.

Continuous ingestion of ds*egfp*-containing diet had no significant impact on nymphal survival relative to blank controls in *S. furcifera* and *L. striatellus*. However, when *S. furcifera* and *L. striatellus* nymphs had been fed on diets containing ds*dib* at concentrations of 0.01, 0.05 and 0.50 mg/ml, significant impacts on survival were observed (Fig. 5A and 5B).

After the experiment, the surviving nymphs were transferred to rice until adult emergence. The mortalities from the termination of experiment to adults emergence were 8.4% (±1.3%, SE), 7.6% (±1.4%), 15.3% (±1.5%), 17.4% (±1.8%), and 21.5% (±2.2%) respectively for *S. furcifera* on normal, *egfp*-dsRNA-, and 0.01, 0.05 and 0.50 mg/ml ds*dib-*containing diets; and were 9.1% (±1.5%), 11.8% (±1.3%), 14.9% (±1.6%), 20.2% (±1.7%), and 28.9% (±2.3%) respectively for *L. striatellus* nymphs. For both planthopper species, the mortalities at 0.50 mg/ml were significantly higher than those on normal and *egfp*-dsRNA-containing diets (one-way ANOVA analysis, P<0.05, df = 4,20).

### Ingestion of dsRNA on Nymph Development

The development of *S. furcifera* and *L. striatellus* nymphs that were fed ds*dib*-containing diet was significantly delayed. More than 90% surviving planthoppers on normal and *egfp*-dsRNA-contained diets developed to the fourth (for *S. furcifera*) or fifth (for *L. striatellus*) instar. In contrast, only 83.7%, 64.6% and 41.3% of *S. furcifera* survivors reared on diets containing *Sfdib*-dsRNA at concentrations of 0.01, 0.05 and 0.50 mg/ml reached the fourth-instar (Fig. 5C). Likewise, 74.3%, 63.2% and 37.8% of *L. striatellus* individuals reared on diets containing *dsLsdib* at concentrations of 0.01, 0.05 and 0.50 mg/ml reached the fifth instars (Fig. 5D). In *S. furcifera*, the time required to develop from the 2^nd^ to the 4^th^ instar on normal diet, ds*egfp*-diet and diets containing ds*Sfdib* at the concentration of 0.01, 0.05 and 0.50 mg/ml were 7.0 (95% confidence interval 6.2–7.8), 6.7 (5.7–7.7), 8.0 (7.3–8.7), 8.4 (7.5–9.3) and 9.2 (8.3–10.1) days respectively. Nymphs fed the normal or dsegfp-containing diets had significantly shorter developmental times than nymphs fed with 0.50 mg/ml ds*Dib* (P<0.05, df = 4,20) (Fig. 5E). Similarly, the average duration of *L. striatellus* fourth-instar stages on normal, ds*egfp*- and the diets containing ds*Lsdib* at the concentration of 0.01, 0.05 and 0.50 mg/ml were 4.9 (4.6–5.2), 5.0 (4.4–5.6), 5.6 (5.0–6.2), 5.8 (4.8–6.8) and 6.5 (5.8–7.2) days respectively. Nymphs fed with diet containing 0.50 mg/ml of ds*Dib* took significantly longer to develop than nymphs fed either normal or ds*egfp*-diet (P<0.05, df = 4,20) (Fig. 5F).

### Rescue Experiment

**Figure 6:**
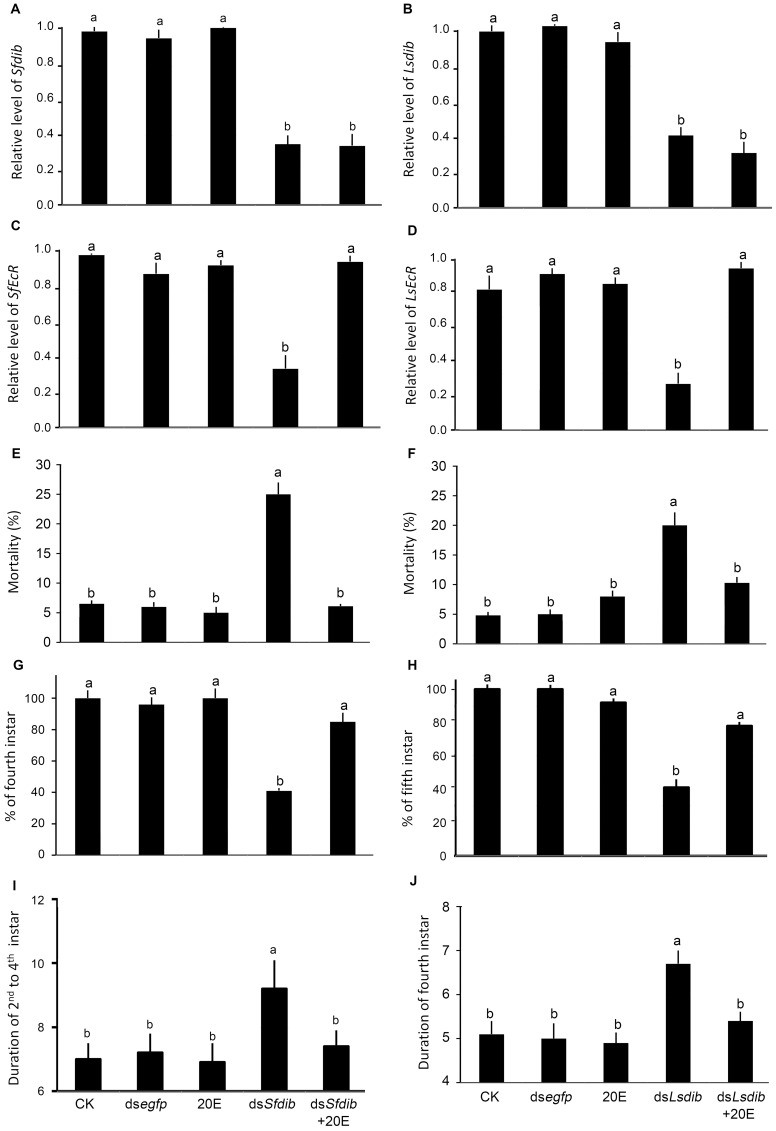
Effects of ds*dib*, 20E, and 20E+ds*dib* ingestion on *S. furcifera* (A) and *L. striatellus* (B). The nymphs were continuously exposed to dsRNA, 0.5/mL 20E and 0.5 mg/mL 20E+dsRNA. The relative expression levels of *dib* and *EcR* genes, mortality and duration were measured. The values represent averages with vertical lines indicating SE. Lines with the same lowercase letters are not statistically different at P = 0.05.

Ingestion of 20E did not affect the expression level of *dib* in either *S. furcifera* or *L. striatellus* nymphs. In contrast, 20E ingestion restored the mRNA expression levels of *SfEcR* and *LsEcR* similar to those of controls (Fig. 6C and 6D). Moreover, 20E ingestion almost completely overcame the negative effects on the nymphal survival (Fig. 6E and 6F) and development in ds*dib*-treated *S. furcifera* and *L. striatellus* nymphs (Fig. 6G to 6J).

## Discussion

Oral delivery of dsRNA has a potential practical application as an insecticide in the field. Moreover, it is more convenient and easy to manipulate than microinjection [8]. Oral delivery of *in vitro*-synthesized dsRNA has previously been used to induce gene silencing in a variety of pest insects in Lepidoptera [39]–[42], Coleoptera [43]–[47], Hymenoptera [48], Diptera [44], [49]–[53], Hemiptera [8]–[13], [16], [44], [54]–[56], Orthoptera [57] and Isoptera [58]. In the present paper, we cloned and characterized putative *disembodied* (*dib*) genes in two planthoppers *S. furcifera* and *L. striatellus*, and tested whether oral delivery of ds*dib* effectively down-regulated the target genes.

### Characterization of Sfdib and Lsdib Proteins

We provided here three lines of experimental evidence to support the hypothesis that the putative *Sfdib* and *Lsdib* encoded functional Dib proteins. Firstly, predicted SfDib and LsDib showed typical protein domains, and temporal and spatial expression patterns associated with ecdysteroid biosynthesis. SfDib and LsDib have five insect conserved P450 motifs, i.e., Helix-C, Helix-I, Helix-K, PERF and heme-binding motifs. Similar structural characters have been documented in Dib-like proteins from other insect species of diverse orders [17]–[27]. Moreover, the N-termini of SfDib and LsDib have a mitochondrial targeting sequence and possess two positively charged residues near the heme-binding domain, two important features of mitochondrial P450 enzymes. Consistent with the structural characters, mitochondrial localization of Dib was observed in *dib* transfected *Drosophila* S2 cells [59]. In addition, 22-hydroxylase activities are detected in mitochondria prepared from *Locusta* [60] and *Manduca* [61].

Our qPCR results revealed that *Sfdib* and *Lsdib* showed expression peaks in late instar stages. In contrast, their expression levels were lower in the newly-molted nymphs. In *N. lugens* [62] and *S. littoralis* [26], the level of E and 20E showed a peak in the later instar stage. Thus, it can be speculated that the temporal expression pattern of *Sfdib* and *Lsdib* coincides with E and 20E titers in the haemolymph. Similarly, the variations of expression levels of *Bmdib* and *Msdib* were correlated with the changes in E and 20E titers in the haemolymph of *B. mori* [20] and *M. sexta* [21]. Moreover, we found that *Sfdib* and *Lsdib* were expressed at higher levels in the thoraces where prothoracic glands were located, compared to the mRNA levels in corresponding heads or abdomens. The spatial expression patterns of *Sfdib* and *Lsdib* are consistent with their function in the paired prothoracic glands. Similarly, *dib* genes were observed to be expressed in the prothoracic glands of various insect species such as *D. melanogaster* [18], [19], *B. mori* [20] and *M. sexta* [21]. In addition, *Sfdib* and *Lsdib* were slightly but distinctly expressed in the head and abdomens. Similarly, even though *dib* had highest expression level in the prothoracic gland of *M. sexta* fifth-instar larvae, substantial expression was observed in the fat body, midgut, ganglia, Malpighian tubules, and epidermis, in descending order. Moreover, *dib* was also expressed in several pupal tissues and in the adult ovaries of *M. sexta* [21]. In *S. littoralis*, *dib* was also found in midgut, brain, fat body, epidermis and Malpighian tubules in the sixth instar larvae [26]. These tissues may operate as secondary sources of ecdysteroids by catalyzing terminal hydroxylation from late intermediates, and may provide extra-prothoracic gland ecdysteroids for local effects such as paracrine and autocrine during larval development and metamorphosis.

The second line of experimental evidence was that RNAi-mediated depletion of *Sfdib* and *Lsdib* caused phenotypic defects similar to insects whose ecdysteroid synthesis had been disturbed [14], [15], [24] or whose ecdysteroid-mediated signaling had been inhibited [63], [64]. RNAi-mediated knockdown of *dib* reduced *ecdysone receptor* gene *EcR* expression at mRNA levels in both *S. furcifera* and *L. striatellus* nymphs. Since EcR is a nuclear receptor of ecdysteroids [65], our results raise the possibility that dietary introduction of *Lsdib*-RNA inhibit ecdysteroid signaling pathway. Since mutations in and RNAi against *EcR* cause phenotypic defects and lethality in *T. castaneum* [63], and in *L. striatellus* and *N. lugens* [64], we observed the influence of *Lsdib*-dsRNA ingestion on nymphal development to test the possibility. As expected, RNAi-mediated depletion of *dib* genes caused nymphal lethality and developmental delay in both *S. furcifera* and *L. striatellus* species.

The third line of evidence was that ingestion of 20E almost completely rescued *EcR* expression, and overcame the negative effects on the survival and the development in ds*dib*-treated *S. furcifera* and *L. striatellus* nymphs. This suggested that ds*dib* ingestion by *S. furcifera* and *L. striatellus* negatively affects ecdysteroidogenesis, and subsequently down-regulates *EcR* expression in the two planthoppers. Similarly, knockout and knockdown of Halloween genes caused a decrease in ecdysteroid titers in other insect species [18], [19], [66]–[70]. Thus, we provided the third line of evidence to support the hypothesis that putative *Sfdib* and *Lsdib* genes encode functional Dib proteins that play a critical role in ecdysteroidogenesis in *S. furcifera* and *L. striatellus*.

### Ingestion of dsRNA Showed Potential Application of Pest Management

In this study, the effects of orally delivered dsRNA on *dib* and *EcR* transcript levels in *S. furcifera* and *L. striatellus* were dose-dependent, with higher concentrations of ds*dib* causing higher negative effects. Similar results have also been documented in *Aedes aegypti* [51] and *N. lugens* [71]. In previous experiments in *D. melanogaster* and three other non-dipteran insect species, it was found that oral delivery of dsRNA at doses higher than 0.5 mg/ml was no more effective at inducing RNAi and killing the target insects [44]. Similarly, several studies have also observed that increasing the concentration beyond an optimal dose does not improve the extent of RNAi, although the optimal concentration may vary for the species, mode of delivery, and the life stage [55], [57], [72]. Thus, in the present paper, we tested the negative effects of dietary ingestion of ds*dib* at the concentration of 0.01, 0.05 and 0.50 mg/ml. We did not find the optimal concentration of ds*dib* in the present paper, and RNAi-mediated knockdown of *dib* genes was thus incomplete in *S. furcifera* and *L. striatellus*. Even so, our data were comparable to gene silencing levels observed in many other insects that have been fed dsRNA, where the extent of RNAi-induced silencing typically ranged between 40% and 60% [9], [41], [49], [51], [52], [54], [58]. Thus, our results in this study offer two possible applications. Firstly, an oral dsRNA delivery method in pest will facilitate the development of higher throughput RNAi screens in *S. furcifera* and *L. striatellus*. Such high throughput screens may identify new targets for control technologies. Secondly, the RNAi-induced effects in planthoppers could serve as a possible method for pest control.

It is widely recognized that two factors are critical for dsRNA-based pesticides, namely effectiveness and dsRNA delivery method [73]. As for effectiveness of dsRNA, our results showed that 5- or 6-day’s ingestion of ds*dib* by second-instar *S. furcifera* and fourth-instar *L. striatellus* nymphs killed approximately 15%–25% planthoppers during the experiments, and caused a further 15%–20% of individual to die after the feeding experiments. RNAi depletion of *dib* gene should also affect embryonic and nymphal development, and adult reproduction [18], however, here we only tested the negative effect of ds*dib* ingestion on nymphal development. We need to perform further experiments in future to evaluate the influence of ds*dib* on adult reproduction and developing embryos.

As for dsRNA delivery, it has been reported that transgenic rice expressing insect-specific dsRNAs mediate RNAi-based knockdown of the target genes in *N. lugens* insects, although the exact dsRNA concentration in rice was not measured [10]. *S. furcifera* and *L. striatellus* deposit their eggs in the leaf sheaths of rice plants, and both nymphs and adults feed on rice phloem sap. Considering that a transgenic rice may provide the insecticidal dsRNA to pests in a stable and potent form [10], [40], [43], [74], it can be expected that the transgenic rice may act as a potential ovicide through planthopper embryonic development stage, as well as a stomach toxin to whole nymphs and adults. Thus, the transgenic rice expressing dsRNAs of Halloween genes such as *dib* provides a potential application for planthopper control and deserves further investigations.
